# Diagnostic and Therapeutic Approach to a Neuroenteric Cyst in the Lumbar Spine

**DOI:** 10.7759/cureus.71048

**Published:** 2024-10-07

**Authors:** Asim Mehmood, Muhammad Nadeem, Rida Inam, Rukhsana Parveen

**Affiliations:** 1 Internal Medicine, Shifa College of Medicine, Islamabad, PAK; 2 Neurosurgery, Shifa International Hospital, Islamabad, PAK

**Keywords:** case report, intradural cyst, lumbar pain, neuroenteric cyst, spinal anomalies, spine surgery

## Abstract

We present a case of a 48-year-old woman who began experiencing excruciating lower back pain that radiated down her right leg and got worse in 15 days. An intradural cyst at L2, which compressed the conus medullaris and cauda equina, was found to be T2 hyperintense and T1 isointense on magnetic resonance imaging (MRI). Upon histological inspection, a well-defined ovoid cyst was observed and identified as a neuroenteric (NE) cyst, which was somewhat brighter than cerebrospinal fluid (CSF). Cyst excision and L2 laminectomy provided her with significant pain relief and a smooth recovery. She had completely recovered from her symptoms by the two-week follow-up. This case demonstrates the importance of MRI in diagnosing NE cysts and the efficacy of surgical removal in producing favorable results.

## Introduction

Neuroenteric (NE) cysts comprise 0.7% to 1.3% of all spinal tumors and are uncommon developmental anomalies. Associated cysts are located in the mediastinum and abdomen in addition to the spine. Ventral to the spinal cord is where most NE cysts are found. The intradural-extramedullary compartment contains about 95% of NE cysts; the remaining 5% are found in extradural and intradural locations [1]. These cysts are congenital lesions that resemble the mucosa of the gastrointestinal tract and are lined by non-ciliated, simple, or pseudostratified cuboidal or columnar epithelium that produces mucin [2]. The cyst is a representation of endodermal tissue that has extended or become enclosed within the ectodermal central nervous system. These days, clinicians have the opportunity to diagnose these disorders prior to surgery using magnetic resonance imaging (MRI). When a patient has symptoms, surgical resection continues to be the mainstay of treatment. During surgery, the main goal is to obtain sufficient surgical exposure in order to fully visualize the interface between the cyst and the spinal cord [3].

## Case presentation

A 48-year-old female with no known comorbidities presented to the outpatient department with a complaint of lower back pain for the last 15 days. The pain was sharp in character and sudden in onset but had been increasing in intensity each passing day, radiating to the right leg, and was not associated with any numbness, weakness, or signs of cauda equina syndrome. Pain was relieved somewhat with non-steroidal anti-inflammatory drugs (NSAIDs), but there was no complete relief, and no aggravating factors were noted. On the pain scale, she rated it as 9 out of 10. There was no associated trauma history. The patient's appetite was normal, but she complained that her sleep had decreased over the past two weeks. Her family history was positive for hypertension and myocardial infarction. Her past surgical history included a cholecystectomy performed four years ago, and her medical history was otherwise insignificant.

On general physical examination, the patient appeared healthy, with normal vital signs, sitting comfortably in a chair, fluent speech, normal pupils, and extraocular movements. There was no fasciculation or wasting on motor examination of the lower and upper limbs. Gait and tone were normal, and power was 5/5. Deep tendon reflexes of the lower limbs were increased but normal (2+) and normal for the upper limbs. The straight leg raise test was positive in both legs, with a value of 35 degrees for the right leg and 60 degrees for the left leg. On sensory examination, temperature, light touch, crude touch, and vibration senses were intact in all dermatomes of the upper and lower limbs.

**Figure 1:**
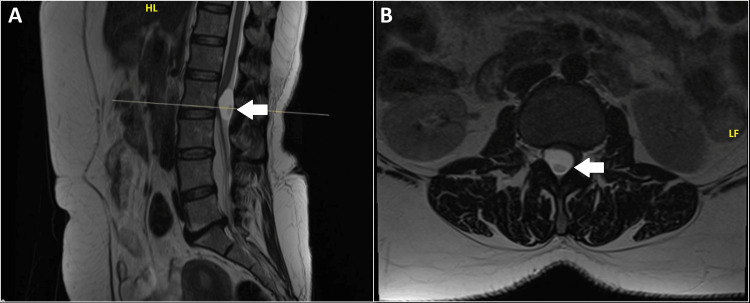
Sagittal (A) and axial (B) contrast-enhanced MRI views of the lumbar spine show a well-defined, ovoid, non-enhancing extra-medullary intradural lesion at the level of the L2 vertebral body. Arrows in the images indicate the lesion, which significantly compresses the conus medullaris and cauda equina roots, predominantly affecting the anterior and left side. HL: head-left, LF: left-hand.

A multiplanar, multisequential MRI of the lumbar spine with contrast was requested, which revealed mild straightening of the lumbar spinal curvature with grade 1 retrolisthesis of L5 over S1 and reduced intervening disc space. A well-defined, ovoid, T1 isointense and T2 hyperintense extramedullary intradural signal abnormality measuring 1.3 cm × 1.4 cm × 2.8 cm (AP × TR × CC) at the level of the L2 vertebral body was found, compressing the conus medullaris and cauda equina nerve roots anteriorly and towards the left (Figure 1). It had a slightly brighter signal than cerebrospinal fluid (CSF). Differentials of an arachnoid cyst or a NE cyst were suspected.

**Figure 2:**
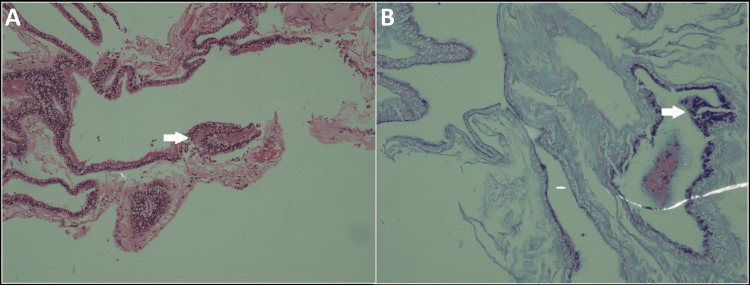
H&E-stained slide at 20× magnification shows (A) a cyst wall lined by a single layer of ciliated cuboidal to columnar epithelium, with cilia indicated by the white arrow, resting on a collagen layer. The PAS-AB special stain (B), at 20X magnification, highlights goblet cells within the cyst wall. H&E: hematoxylin and eosin, PAS-AB: periodic acid-Schiff-Alcian Blue.

Surgery was planned, and the patient was prescribed thiocolchicoside, pregabalin, and multivitamins for a week. Her preoperative tests were sent, and their results came back within normal ranges, and she was declared fit for surgery. She underwent L2 laminectomy and excision of the intradural lesion. The dura was secured and repaired. The excised specimen was sent to the histopathology lab for biopsy, which confirmed it as an NE cyst with a wall lined by a single to multilayer of ciliated cuboidal to columnar cells having round nuclei, vacuolated cytoplasm and an intact basal membrane. The underlying stroma was hyalinized with mild inflammation (Figure 2). Immunostain carcinoembryonic antigen was positive in the lining epithelium. No evidence of atypia, mitosis, or invasive components was seen. Furthermore, the specimen revealed a few pus cells. It tested negative for acid-fast bacilli, yeast, epithelial cells, and gram-positive and gram-negative cocci and rods.

The patient was stable after surgery and experienced pain relief. Her hospital stay was uneventful, and she was mobilized on postoperative day two. Her surgical wound was dry and healthy, and there was no CSF leak from the wound. The patient was discharged from the hospital with prescribed medications, including NSAIDs, lactulose syrup, omeprazole, and multivitamins for two weeks, and instructions to follow a protein-rich diet and avoid climbing stairs, lifting heavy weights, sitting without back support, and constipation. She was advised to follow up after two weeks and was counseled about red flags and to report to the emergency department in case of severe uncontrolled pain, vomiting, or fever. On follow-up after three weeks, the patient was found to be clinically stable.

## Discussion

Spinal NE cysts typically appear in the second and third decades of life and are rare congenital lesions with a slight male preponderance. The cervicothoracic region is where they are frequently observed. During the third week of embryogenesis, they arise from an incomplete separation of the embryonic notochodal plate and endoderm. There have been reports of neural tube cysts associated with spina bifida, spinal dysraphism, and Kippel-Feil syndrome. Additionally, correlations with gastrointestinal, renal, cardiac, and cutaneous anomalies have been documented [4].

Clinical symptoms, which include pain and, depending on the level involved, motor or sensory deficits, are typically a result of meningeal irritation. Lesions affecting the cervicothoracic region are typically associated with myelopathic symptoms, whereas lesions affecting the cervical and lumbar regions are associated with radicular symptoms. Depending on the patient's age, the size, and the location of the lesion, NE cysts can present in various ways. As with our patient, the most common and initial symptom is usually back pain. Compression of the spinal cord and nerve roots is the primary cause of pain, which can be either localized or radicular [5].

A few individuals with NE cysts exhibit sporadic symptoms akin to those of multiple sclerosis. The cause of intermittent symptoms is likely a thin-walled spinal canal cyst that progressively grows larger due to secretion from the cyst wall and osmotic retention of CSF and shrinks smaller due to tiny subarachnoid space ruptures and mucin reabsorption by the cyst wall. Adults frequently exhibit symptoms of spinal cord compression, which can happen after minor injuries. Neurological symptoms may occasionally appear suddenly as a result of spontaneous bleeding into the cyst or an increase in cyst size brought on by an intracystic fluid buildup [6]. The most reliable neuroimaging technique for diagnosing this condition is MRI. Studies have shown that the absence of a mural nodule and the cyst wall's lack of contrast enhancement on MRI help differentiate this disorder from other malignant lesions. Moreover, MRI provides detailed information about the location of the cyst within the neuraxis, the extent of compression on the spinal cord, and whether an abdominal or posterior mediastinal cyst coexists. Additional research has revealed that MRI patterns, cyst content, and CSF may all be similar, or they may all be proteinaceous with a shortened T1 relaxation time and intense appearance on T1-weighted images [7]. However, a computerized tomography (CT) scan is helpful in eliminating any associated bony vertebral malformations, such as segmentation anomalies, butterfly vertebrae, and vertebral bony clefts, which may coexist in approximately 50% of cases. Wilkins and Odom divided neurenteric cysts into three types based on histology. Type A cysts are made up of cuboidal or columnar cells that are either ciliated or non-ciliated and rest on a type-4 collagen basement membrane. All the components of type-A cysts, plus bone, fat, cartilage, and/or lymphatic tissue, make up type-B cysts. Together with type-A contents, type-C cysts also include glial or ependymal tissue [8].

NE cysts within the spinal canal are best treated with complete surgical excision, avoiding neurovascular damage. Anterior, posterior, and lateral techniques are some of the approaches that have been documented in the literature. Since the posterior approach is linked to fewer complications, it is preferred [9]. While total removal is ideal, it might not always be achievable. Because the wall of the cyst is attached to the spinal cord, Kozak et al. recommended an anterior central corpectomy procedure for anterior neurenteric cysts of the cervical spine. According to them, it was technically challenging to completely remove the cyst during a posterior laminectomy without using spinal cord manipulation. Intramural cysts, restricted access, abnormalities of the bones, and adhesions to the spinal cord are some factors that make excision challenging [10].

There is a chance that the complete removal of intramedullary neurenteric cysts will result in irreversible brain damage. Therefore, as some case series have shown, a planned subtotal excision may be appropriate in such circumstances. Untreated intraspinal NE cysts have a poor natural history. The appropriate course of action for NE cysts is total surgical excision. Even after long-term symptom relief, these cysts can recur, so annual MRIs and a lifetime of follow-up are necessary [11].

## Conclusions

The significance of taking NE cysts into account when making a differential diagnosis for persistent lumbar pain is demonstrated by this case. A compressing intradural NE cyst at L2 was discovered by MRI. Recovery and pain were significantly reduced after the surgery. This case highlights the importance of MRI in providing precise diagnoses and the efficacy of surgical intervention in treating uncommon spinal deformities.
